# Hospital and Institutional News

**Published:** 1919-09-13

**Authors:** 


### HOSPITAL AND INSTITUTIONAL NEWS.

### POST-GRADUATE STUDY.

THE Fellowship of Medicine has undertaken to carry on its work on behalf of post-graduate teaching in London. With the hearty co-operation of the whole profession this valuable service is being reorganised for the coming winter. A series of special lectures will be given at the Royal Society of Medicine, beginning on September 15. The following is the programme as it stands at the moment. September 15, at noon, by Mr. W. H. Trethowan, "Feet: Mechanical Disabilities"; September 17, at noon, by Mr. L. B. Rawling, "Surgery of the Skull and Brain.—I."; September 22, at noon, by Mr. Trethowan, "Feet: Paralytic Deformities and Disabilities"; September 23, at noon, by Dr. Hutchison, "Chronic Diarrhœa, its Varieties and Treatment"; September 24, at noon, by Mr. Rawling, "Surgery of the Skull and Brain.—II."; September 25, at noon, by Mr. W. H. McMullen, "Some Common Disorders of the Fundus Oculi"; October 1, at noon, by Dr. Eric Pritchard, "The Causation and Treatment of Rickets."

### DOCTORS FOR THE ARMY.

THE War Office is prepared to accept the services of medical practitioners under a new contract which is an improvement on the old, about which many complaints have been made recently. The period of service is reduced from one year to six months. The pay is increased from £550 as an inclusive rate to £600 for lieutenants and £650 for captains with allowances. There will be specialists' or charge pay when the officer holds a position for which the issue of such is authorised, and the new contract is expected to secure many of the younger men.

### NEW REGULATIONS FOR PHARMACEUTICAL QUALIFICATION.

THE new regulations made by the Pharmaceutical Society of Great Britain regarding candidates who register as students after December 31, 1919, have now been published. The examination will now be divided into two parts: the first part of the qualifying examination may be taken only after the student has become registered as such and gone through a specified course in chemistry, botany, and physics. The second part of the examination may be taken after the student has passed the first and has spent 4,000 hours, in a period of not less than two years, in a retail chemist's shop under the direct supervision of a pharmacist. Special provision is made for certain exemptions in favour of those who have served with H.M. Forces so that they shall not suffer hardship by the new arrangements for qualification. It is to be noted that the practical training in dispensing may now only be obtained by serving in a chemist's shop, and apparently no provision is made for accepting the training received in the dispensary of a hospital where, in many cases, there are exceptional facilities for giving a student a thorough knowledge of the preparation of galenicals and of dispensing prescriptions. This seems a pity since, in view of the Ministry of Health developments, there is likely to be an increased demand for pharmacists with special knowledge of making preparations on a large scale for hospital requirements.

### BEE AND WASP STINGS.

IN a recent letter to the *Times*, Mr. Edward R. Speyer, M.A., F.E.S., investigator of diseases of trees at Oxford University, shows that the sting of the bee is decidedly acid, while that of the wasp is distinctly alkaline. In either case the choice of a neutralising agent then becomes obvious, and in the instance of wasp stings the customary prompt application of the blue-bag, ammonia, or carbonate cannot be expected to produce the required beneficial effect. There is some evidence that these stings are not simple acid and alkaline irritants, easily and directly to be neutralised, but it is stated that, after extracting the stings of wasps, immersing them for a short time in an acid, no ill effects result when they are driven deeply into the flesh. By experience we know that application of an acid, such as vinegar, or acid vegetable or fruit juices (onion, lemon, etc.), will prevent extension of inflammation, but it is doubtful whether this simple knowledge is as widely distributed as it might be. A wasp sting in the mucous membrane of the mouth may lead to most serious results, and if the direct application of acid to the locality keeps the swelling down a number of lives might be saved each year. Simple washing or gargling, or even submucous injection, might be resorted to.

### FEES FOR PUBLIC VACCINATION.

THE Association of Public Vaccinators of England and Wales has justified its existence in a number of ways. Not the least is its activity in defence of vaccination *per se* for many years. During June Major Astor received a deputation stating the case for improved fees and conditions for public vaccinators, and calling for a revision of the Act of 1907, which has been the cause of much neglect of vaccination by substituting a statutory declaration for the certificate of conscientious objection. This view was sympathetically received, and will no doubt receive careful consideration. In July again a letter regarding an increase of fees was addressed to the Ministry, and, in reply, it was stated that the Minister has it in contemplation to modify the existing Order by raising to 5s. the present minimum fee of 2s. 6d. or 3s. 6d. for primary vaccination performed at the residence of the person.

### THE CROWN AND LAMBETH.

MUCH has been done in Lambeth by the Crown, as owners of the Duchy of Cornwall Estate at Kennington, to provide decent housing, whilst at Stamford Street a whole street of houses were converted into flats. There are, however, at Kennington, on what is known as the Regency Square area, hundreds of derelict houses. They were closed just before the War with the idea of extending the scheme of rebuilding. Dr. Priestley considers that the time is ripe to press the Duchy of Cornwall to take this rebuilding scheme in hand at once, and, should this be done, it would soon provide accommodation for hundreds of people. The Ecclesiastical Commissioners had to deal with a very bad area in the notorious New Cut, but they tackled the problem thoroughly, and where once were old broken-down houses in courts, alleys, and *culs-de-sacs*, are now neat, two- and three-storeyed houses. But the Ecclesiastical Commissioners also have an estate ripe for development at Brixton, and Dr. Priestley considers that they too should be asked to proceed.

### HOUSING IN LAMBETH.

Dr. John Priestley, Medical Officer of Health for the borough of Lambeth, has presented to his Council a most interesting report on the housing question, paying special attention to the remedial work that has been done during the last twenty years. The borough, though not one of the largest in London, extends from the river-side to the Crystal Palace. The oldest part is at the river-side, with many old houses, which have for generations been the abode of the very poorest of the population. So far as the Council itself is concerned, Dr. Priestley does not advocate the adoption of any housing scheme for the river-side part, and in this policy he is right, because vast private ownerships are prepared, as soon as they can, to go on with their housing schemes. Outside the congested area, at West Norwood there is, however, building land available, and, on his suggestion, the Council has decided to acquire the Holderness House Estate at West Norwood on which to build some 130 dwellings. In other parts it is proposed to convert a number of empty houses into flats.

### THE HOUSE THE WORKERS REALLY WANT.

Those conversant with the housing question in large towns realise that there is a portion of the population to which decent housing does not appeal. Dr. Priestley points out that these "undesirables" seem to call for special treatment at the hands of the central authority, the London County Council. "Cleanliness by Act of Parliament is not to-day a practical suggestion," he writes, "and even the placing of baths in all dwelling houses will not necessarily ensure their use as such, if we may judge by the number of baths reported to be at present actually doing service as receptacles for coals, dirty clothes, old lumber, etc." In the inner wards, Dr. Priestley states, no new model dwellings should be allowed to be built, because of the crowded population. The real demand of the working classes in the borough was, generally speaking, for cottage property or for joint occupation by not more than two families, but these houses should be self-contained.

### A VISIT TO BUDA-PESTH HOSPITALS.

After a visit to Buda-Pesth in July Dr. Hector Munro and Dr. Hilda Clark have sent a brief description of the condition of the hospitals in that city to the *Nation*. During his visit, which occurred while the communists were in power, Dr. Munro found the food supply "fair," but decreasing because the country people declined to sell their produce to the city. They disliked the party in power, and wanted to exchange their produce for goods, but no goods, only "money" of dubious value, were to be obtained. The hospitals chiefly lacked rubber, anæsthetics, and most drugs, and for the past three months all dressings had been made of paper. In August four trucks of dressing and equipment arrived from Vienna, but the Hungarians had not been able to obtain any rubber, anæsthetics, morphine, or soap. Linen was so scarce that "in some hospitals the patients were without sheets," and "nowhere could the linen be changed often enough for the essential needs of cleanliness." This difficulty is complicated by "the shortage of coal, which prevents adequate disinfection." The visitors learned that the mortality rate had risen considerably in two maternity hospitals, where, too, the lack of general anæsthetics had been severely felt. The lack of linen has meant that the baby-clothes and napkins have to be made of paper. These garments only survive two washings; the irritation caused to the skin becomes serious through the absence of "any suitable vaseline" or other ointment. To compare this picture with that of the London hospitals is to note the difference between defeat and victory; but the price of victory too is extremely dear, as we shall all discover during the coming winter.

### INCREASED COST OF LUNATICS.

The London County Council has notified the Metropolitan Boards of Guardians that after October 1 the weekly cost of lunatics in their institutions will be increased to 26s. 10d. per patient. This is the third increase in a year, twelve months ago the cost being 16s. 4d. The rise, therefore, is one of 10s. 6d. each patient. The latest increase, the London County Council Comptroller states, is necessary in order to meet the increases in the rates of pay of the staff at the mental hospitals. Happily, on this occasion ample notice of the rise has been given the Guardians, so that they can make provision for it in their demands on the Borough Councils, but when the last increase of 3s. per week per patient was demanded it came in the middle of a half-year. The result was that the Guardians had no money to meet it, and at the close of the half-year they found themselves practically in a bankrupt position. The increasing rise of this branch of the public services is a serious matter. Few Metropolitan Boards of Guardians have less than 1,000 mental patients, and at the increase of 10s. 6d. each means an extra demand alone every six months of over £6,000.

### CLERGYMAN AND DYING PATIENT.

IT is happily very rare that any public authority has to chronicle such a serious incident as that reported to the Southwark Board of Guardians at their last meeting. A clergyman doing duty for the chaplain at their Christchurch institution, for which he was being paid, after the evening service was asked to visit for a few minutes a man who had been taken suddenly ill and placed on the danger list. He refused to see the patient on the ground that he was not the chaplain, and the patient died next day without the consolations of the Church. Efforts to obtain another clergyman or minister were unsuccessful. The Guardians have decided to place the facts before the Bishop of Southwark, with an expression of their deep resentment at such conduct. It was the first time in the history of the Guardians that this has occurred, and it is to be sincerely hoped that, as the Chairman (Councillor W. Savage) remarked, it will be the last. Had the Guardians not brought this conduct to the notice of the Bishop of Southwark, it is gratifying to know that the chaplain would have done so.

### A NEW HOSPITAL SECRETARY OF LOUGHBOROUGH HOSPITAL.

FROM seventeen applications for the Secretaryship of the Loughborough and District Hospital and Dispensary the Committee of Management have elected Mr. Frank H. Toone, a local solicitor, at a salary of £75 per annum. Mr. Toone succeeds Mr. T. J. Webb, who is retiring after many years' service. Mr. Toone has already done good local service, and for years has been the Hon. Secretary of the Loughborough and District Royal Lifeboat Association. The Loughborough Hospital fulfils a useful mission in the hospital treatment of the district, although its work is perhaps overshadowed by two greater county hospitals, viz., the Nottingham General and the Leicester Royal Infirmary. The town has a large industrial, and the district a good resident, population. The industries have been fully employed during the war. This means larger financial support for the hospital, and doubtless the Committee and the new Secretary will, if necessary, bring their administration into close touch with the local population. The industrial classes contribute increasingly large proportions of the cost of hospitals and institutions, especially when, in a measure, they share in the management. There is undoubtedly a wide field open to the new secretary, which his local knowledge and professional standing will doubtless enable him to take advantage of.

### THE RETIREMENT OF CANON STEPHENS.

TO continue the work of honorary secretary until the age of 87 is an example of enthusiasm for hospital work which deserves recording. The feat has been accomplished by Canon J. O. Stephens, who is now retiring from that post at Woodhall Spa. "With the exception of the time I was founding the Parish of All Saints, Tooting," he said, in announcing his resignation, "I have practically supervised this hospital as honorary secretary since its foundation some 30 years since." He had been assisted in the course of his work, Canon Stephens added, by the late Hon. Edward Stanhope, Lord Chaplin, the late Lord Alverstone and Mr. Cheney Garfit. The strain of the work at his advanced age has been great, but he is sorry to say good-bye to the management of the hospital which, "as honorary secretary, I have loved, I may truly say, with all my heart." In addition to the ordinary work, Canon Stephens arranged for the reception of Belgian soldiers and later of British officers. The hospital, he must be pleased to note, he leaves without a deficit, notwithstanding the great rise in the price of all provisions, and the increased wages of the staff.

### THE PROBLEM AT PETERBOROUGH.

THE Peterborough War Memorial Hospital Committee has carried its plan for a new hospital a step further by recommending the Building Committee to prepare specifications and to invite designs from architects. This decision means that the plans are to give an air of reality to a much-discussed proposal, and in that way to attract support. It means also that the Memorial Committee, which intends to build a new hospital on the site acquired through the generosity of Mr. J. H. Bunting, thinks that an accommodation can be arranged with the trustees of the present hospital, whose co-operation is desired. The trustees have naturally been cautious in expressing themselves, because the plan for the new hospital is not mature, but their attitude is friendly, as that of the governors is likely to be when the new scheme is far enough advanced to let trustees and governors know to what they will be committed. They do not want, however, to be the basis of the new scheme. They are ready to co-operate when the new scheme is a reality. For if it is going to succeed it must have a vitality of its own, independently of the present hospital. If sufficient funds can be raised to build on the site purchased, then no doubt governors and trustees of the present hospital, which cannot conveniently be extended, would be ready to amalgamate. Except for Mr. Bunting's gift, the scheme for a War Memorial Hospital has found at present an uncertain amount of support. If it succeeds, it will provide 100 beds. There are sixty in the present hospital.

### THE MILITARY OCTOPUS AND STOCKPORT SCHOOLS.

WHEN the demand for beds for the wounded was at its height a raid was made upon the schools, and we did our best at the time to point out the disadvantages attending their conversion. In some cases other buildings were chosen. In others most of the schools were taken, in consequence of which those few left alone were compelled to work double shifts. The education and health of children were alike injured; but unfortunately the military have not been nearly as prompt to evacuate the schools as they were to occupy them. A glaring case has been reported from Stockport, where six big schools were commandeered. Up to a fortnight ago only two of the six schools had been returned. The time which should have been ample for clearing them has been occupied with correspondence. The education committee has been referred from department to department, and the chief thing accomplished has been delay. In despair the education committee is moving its equipment into all the schools so that they may be ready for use at the beginning of term, September 8. In the same connection it is recorded that the military authorities are still occupying Stepping Hill Infirmary, where the military patients have been reduced to one. A kindred difficulty created by the War Office is its procrastination in the repair of the playgrounds, which has been urged again and again since April, and remained unsettled at the end of August.

### THE FUTURE OF SWANSEA HOSPITAL.

An alluring prospect is held before the Swansea Hospital, which is on a somewhat cramped site, and badly needs more beds, a nursing home, and some attached grounds. The house and land known as Parc Wern, which the owner, Miss Dulcie Vivian, has allowed to be used as a Red Cross Hospital during the war, is expected to be closed, and it is believed that Miss Vivian's wish is that it should remain a hospital. If this can be arranged, Parc Wern would be a valuable addition to the Swansea Hospital. The children could be accommodated there, and the big waiting-list reduced accordingly. It would even be possible to sell the present site or use it for casualties and out-patients, and transfer the hospital to Parc Wern if the house there were enlarged. Such a scheme would be costly, but in any case the opportunity, or rather the chance, of using Parc Wern in conection with Swansea Hospital should not be missed.

### GAME FOR CONSUMPTIVE NURSES.

The first in the field with the annual appeal for game is the East Anglian Sanatorium, Nayland, Colchester, where over sixty officers and soldiers are under treatment for tuberculosis. The appeal for game which has been issued reminds the public that the rations are stricter than they were last year, and that Dr. Jane Walker is not permitted to receive the extra meat for them which was granted last year. The patients include nursing sisters, and the occupations of the patients include the making of jewelry and toys. Since German toys are already said to be upon the market, and since the patients include nursing sisters who presumably would not have contracted the disease but for the war, the East Anglian Sanatorium has many claims upon public sympathy, and we hope those enjoying the "open air life" of the moors this season will remember it.

### THE ELUSIVE WAR BONUS.

With the continued rise in prices, committees of institutions are still being pressed to consider the addition of further war bonuses to the salaries of their staffs. Where such committees happen also to be Boards of Guardians, they have to consider the interests of the ratepayers. The war bonus system is unsatisfactory for two reasons. It implies a temporary increase, and therefore is certain to cause trouble when the period for which it was granted shall have come to an end. Secondly, the period itself is difficult to define. So far as prices are concerned, the effect of the war will last for two or three years, and it is doubtful if they will ever fall to their pre-war level. War bonuses, therefore, are bound to last long enough to establish a kind of prescriptive right to permanence. Would it not be better, therefore, to convert many of them now into increases of salary? There is this advantage in the plan: An increase of salary is a solid gain. It satisfies aspirations. This is not so with war bonuses. They are secured by a rise in price. Consequently, a case can always be made for an increase of the bonus. The appetite grows by what it feeds on. When this policy has been adopted, its application has been recommended to the junior members of the staff only. The object in view is to avoid a general increase of war bonus by revising the salaries of the lower-paid officials.

### V.A.D. UNIFORM FOR HEALTH VISITORS.

The Health Committee of the Fulham Borough Council suggests putting the lady health visitors in uniform, having come to the conclusion that this will give them a certain amount of authority in connection with their work. It is suggested that they should be provided with uniform coats and hats similar to those worn by ladies of the V.A.D., with a replica of the Fulham coat-of-arms as a cap badge.

### THIS WEEK'S DRUG MARKET.

Some improvement in business is noticeable, and generally speaking prices are fairly steady. It is as yet too early to see what effect the removal of the import restrictions is going to have on prices, but it is probable that in a number of cases we shall witness a downward movement, although the products of so-called "key" industries will not be materially affected for the time being. Potassium bromide continues to be in short supply and the advanced price is firmly maintained; the future of bromides depends, however, to a large extent on the importation of German bromides. Almond oil is slightly lower in price. Markets of morphine have again reduced their quotations. Atropine is also offered at lower rates. Phenacetin is in better supply in that price tendency is downwards. Santonin has very substantially advanced in value; incidentally it may be remarked that the present price of this drug is nearly one hundred (100) times the figure at which it could at one time be bought. Lithia carbonate is dearer. Glycerin has been advanced in price. Other articles with an upward price tendency include menthol, senna, salicylic acid, cream of tartar, and sarsaparilla. On the other hand, calumba root, benzoic acid, phenacetin, barbitone, and honey tend downwards in price. The Government control on quinine has been removed and it may be taken as certain that prices will advance. Not only has the demand increased, but the market is practically under the control of a Dutch Syndicate.

